# Assessment of Different Feature Extraction Methods for Discriminating Expressed Emotions during Music Performance towards BCMI Application

**DOI:** 10.3390/s23042252

**Published:** 2023-02-17

**Authors:** Mahrad Ghodousi, Jachin Edward Pousson, Valdis Bernhofs, Inga Griškova-Bulanova

**Affiliations:** 1 Department of Neurobiology and Biophysics, Vilnius University, 10257 Vilnius, Lithuania; 2 Jāzeps Vītols Latvian Academy of Music, 1050 Riga, Latvia

**Keywords:** music, emotion, EEG, connectivity, MSC, GC, brain-computer music interface, BCMI

## Abstract

A Brain-Computer Music Interface (BCMI) system may be designed to harness electroencephalography (EEG) signals for control over musical outputs in the context of emotionally expressive performance. To develop a real-time BCMI system, accurate and computationally efficient emotional biomarkers should first be identified. In the current study, we evaluated the ability of various features to discriminate between emotions expressed during music performance with the aim of developing a BCMI system. EEG data was recorded while subjects performed simple piano music with contrasting emotional cues and rated their success in communicating the intended emotion. Power spectra and connectivity features (Magnitude Square Coherence (MSC) and Granger Causality (GC)) were extracted from the signals. Two different approaches of feature selection were used to assess the contribution of neutral baselines in detection accuracies; 1- utilizing the baselines to normalize the features, 2- not taking them into account (non-normalized features). Finally, the Support Vector Machine (SVM) has been used to evaluate and compare the capability of various features for emotion detection. Best detection accuracies were obtained from the non-normalized MSC-based features equal to 85.57 ± 2.34, 84.93 ± 1.67, and 87.16 ± 0.55 for arousal, valence, and emotional conditions respectively, while the power-based features had the lowest accuracies. Both connectivity features show acceptable accuracy while requiring short processing time and thus are potential candidates for the development of a real-time BCMI system.

## 1. Introduction

A Brain-Computer Interface (BCI) enables users to control a computer or any other external device using their brain activity, and the electroencephalography (EEG) technique has been effective in these types of applications due to its high temporal resolution and relatively low cost. BCI can be beneficial in a broad spectrum of clinical and non-clinical applications such as in the rehabilitation of people with various mental and physical disabilities, as well as for gaming, augmented reality applications, and more [1,2]. Likewise, BCI can be used to enhance environmental experiences; for example, a Brain-Computer Music Interface (BCMI) may transform the brain signals into musical parameters.

The first attempt to connect the brain to music was made by Adrian and Matthew as they found a correlation between Posterior Dominant Rhythm (PDR) and reproduced brain signals played over a loudspeaker [3]; this was followed by the creation of the first brain-controlled percussion instrument in 1965 that worked based on PDR signals [4,5]. Similar to the case of BCI, the BCMI applications are advantageous in various fields spanning the entertainment industry and healthcare, such as in music therapy. Several studies have addressed the effectiveness of BCMI applications in music therapy by modulating the affective state of their users based on precise indices related to subjects’ affective state, and by composing pieces of music that directly target the listener’s mood [5,6,7]. However, wider application is limited by the selection of sensitive brain activity markers reflecting emotions that can be assessed accurately and fast, while keeping the system agile to adapt to new users.

Numerous studies have focused on spectral power measurements as a tool to reveal the brain activity behind emotional experiences elicited by listening to music. Power changes in different frequency bands and over multiple brain areas have been reported to be related to changes in arousal and valence corresponding to different emotional states [8,9,10,11,12]. However, a large information flow between different cortical areas occurs during emotional music processing [13], and this should be considered when comparing emotions at a neurophysiological level. Two approaches can be taken—evaluations of functional and effective connectivity. While functional connectivity reports on statistical relationships between the signals recorded from two areas in the brain and has no information about the direction of the information flow or the causal influence of the regions on each other [14], effective connectivity defines the influence that a neural system exerts over other neural systems for performing motor, cognitive, and perceptual functions [15] by examining the complicated causal interactions between brain regions and uni- or bi-directional information pathways.

Connectivity and network analysis were utilized to investigate the co-active and connected areas during different emotions while listening to music. Wu et al. demonstrated an increase in the phase coherence index during music perception [10]. Shahabi and Moghimi used support vector machines on features based on directed transfer function connectivity and reported that the perceived valence of music was positively correlated with the frontal inter-hemispheric flow, and negatively correlated with parietal bilateral connectivity [16]. Varroto et al. demonstrated an increase in brain network connections in healthy subjects while listening to pleasant music compared to unpleasant or silent conditions [17]. Maggioni et al. used Granger causality to show that, in people with Parkinson’s disease, music has the capability of facilitating inter-hemispheric communication and partially normalizes brain networks [18]. Marimpis et al. [19] reported high accuracies in the Arousal–Valence classification using functional connectivity features within the DEAP dataset [20]. Mahmood et al. reported that even a short duration of listening to music can change the functional connectivity in the brain, proportional to the genre of music [21].

Although a vast number of studies have focused on recognition of emotions evoked by music-listening, there is limited number of studies that address the associations between the neural activities of performers and their intentionally expressed emotions while they are performing a piece [22,23]. It has been shown that brain activity during the perception of emotions induced by music-listening differs from that involved in the creative expression of emotion through music performance [24]; moreover, it is possible that music performers experience different emotions than what they are trying to express through the performance [25]. In our previous works [11,12], we demonstrated that both power and effective connectivity patterns differ when professional musicians are instructed to play distressed, excited, depressed, relaxed, or neutrally labeled music pieces.

In the current study, we aim to evaluate the utility of different methods of feature extraction to discriminate between expressed emotions during music performance [11,12] for the development of a real-time BCMI system. Specifically, we (1) focus on power as well as functional and effective connectivity measures—Magnitude Square Coherence and Granger Causality—due to the simplicity of implementation, short computation time, and processing requirements that make the measures suitable candidates for developing real-time systems, and (2) utilize the Support Vector Machine (SVM) to evaluate and compare the performance of the extracted features, as it is one of the most powerful models for multi-class regression and classification while being fast enough for real-time applications [26].

## 2. Materials and Methods

### 2.1. Participants

Ten musicians (2 males, 8 females; age 19–40 years) experienced in piano playing and with at least 5 years of academic training participated in the experiment. The experiment took place over four sessions that were scheduled on different days. Rīga Stradiņš University Research Ethics Committee approved the study (Nr.6-1/01/59), and all participants provided their written consent.

### 2.2. Experimental Design and Procedure

**Figure 1:**
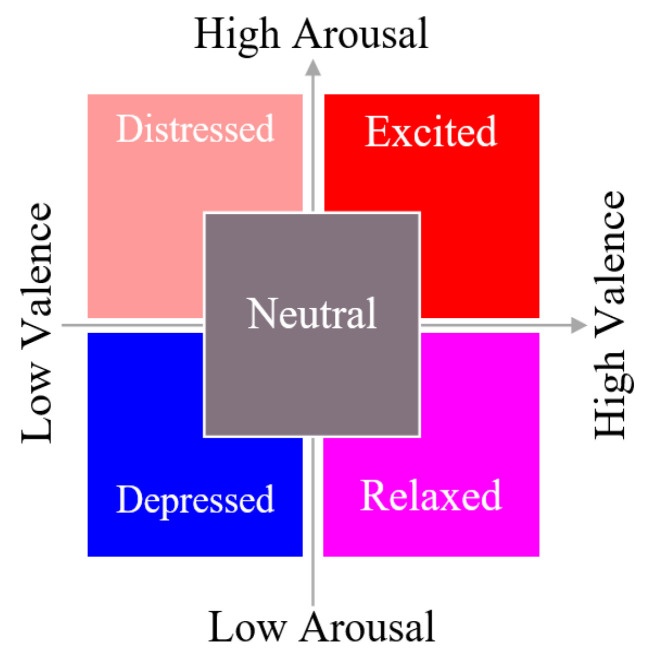
Emotion descriptors used for performance instructions.

Participants received a simple-to-learn short (30 s) musical score produced by the authors [11] and were tasked with making expressive variations upon the material. The detailed description of the procedure is provided in [11]. The music was presented on two pages and used an extended pentatonic scale to circumvent Western functional harmony bias. The first page was to be performed mechanically, in tempo, neutral in expression, and without emotion. The music on the second page was a duplicate of the first page, but the participants were encouraged to alter their manner of play freely in order to express one of five emotions based on a 2D Valence–Arousal model of affective space (distressed, excited, depressed, relaxed, and neutral) (Figure 1).

**Figure 2:**
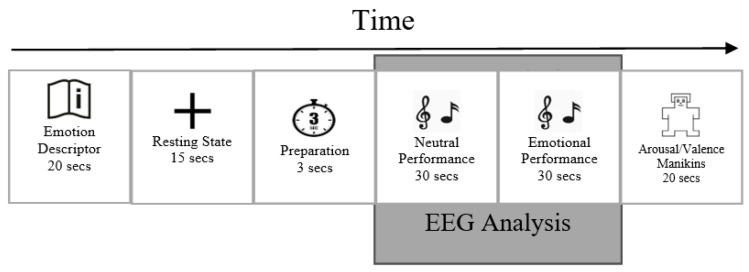
The sequence of a single experimental trial.

The protocol was controlled with the Psychopy stimulus presentation software [27]. The Psychopy stimulus presentation software randomized the order of the five emotions during each recording session and displayed the instructions for each trial. Each participant had a total of 200 trials recorded over the course of 4 days. A scheme of a single experimental trial is depicted schematically in Figure 2.

Before the first recording session, each participant received a thorough explanation of what to expect. Subjects had enough time during the first session to become familiar with the recording sequence and emotional descriptors. Participants were instructed to stay seated at the piano and follow instructions displayed on an eye-level laptop screen.

First, for 20 s, one of five emotion descriptors was shown. Second, during the 15 s of resting state recording, a fixation cross was shown. A countdown notification of the upcoming emotional playing condition that was displayed for 3 s at the end of resting state period, followed by the appearance of the music score. The first page of the music score, instructed to be played in a neutral way, was displayed for 30 s. It was followed by displaying the second page for 30 s while the participants were instructed to perform it while expressing one of the target emotions. After this, on a scale from 1 to 9, the participants rated their performance on the dimensions of valence (from negative to positive) and arousal (from low to high), with 5 serving as neutral on both scales. Participants were asked to submit their scores according to how effectively they believed their performance portrayed the desired feeling rather than on the actual emotions they had experienced. Participants were informed that the audio from the performance could be reviewed by listeners in subsequent steps to make the setting more realistic. After completing 5 consecutive trials, participants were given a short rest period before continuing.

### 2.3. EEG Acquisition

EEG signals were acquired using an Enobio 32 device, with a 32-electrode cap arranged according to the International 10–20 system. Common Mode Sense (CMS) and Driven Right Leg (DRL) electrodes were connected to the right earlobe to provide the electrical grounding. Signals were monitored and recorded at a sampling frequency of 500 HZ using the hardware’s native signal acquisition software Neuroelectric Instrument Controller v.2.0.11.1 (NIC), and a notch filter was applied by the software to remove power line noises.

### 2.4. EEG Preprocessing

EEG data was preprocessed automatically in MATLAB using EEGLAB toolbox [28]. First the Automated Artifact Rejection function in EEGLAB was used, and parts of the raw EEG data with poor quality were removed, which also resulted in removing some of the channels. Then data was filtered using a zero-phase bandpass FIR filter between 0.5 to 45 Hz and re-referenced to the mean of T7 & T8 channels. Further, the retained artifacts such as eye blinks and muscle and heart electrical activity, as well as any other activity not originating from the brain, were removed by applying an Independent Component Analysis (ICA) and ICLabel plugin in EEGLAB.

In every single trial, the signals recorded during 30 s of neutral baseline performance and 30 s of emotional performance (distressed, excited, depressed, relaxed, and neutral) were extracted resulting in two separate matrices of 2000 EEG segments (data from 10 participants across 4 days and 50 piano-playing excerpts per session) for emotional playing (400 observations per each emotion) and 2000 EEG segments for the corresponding neutral baseline, and both matrices were treated similarly in the next steps.

**Figure 3:**
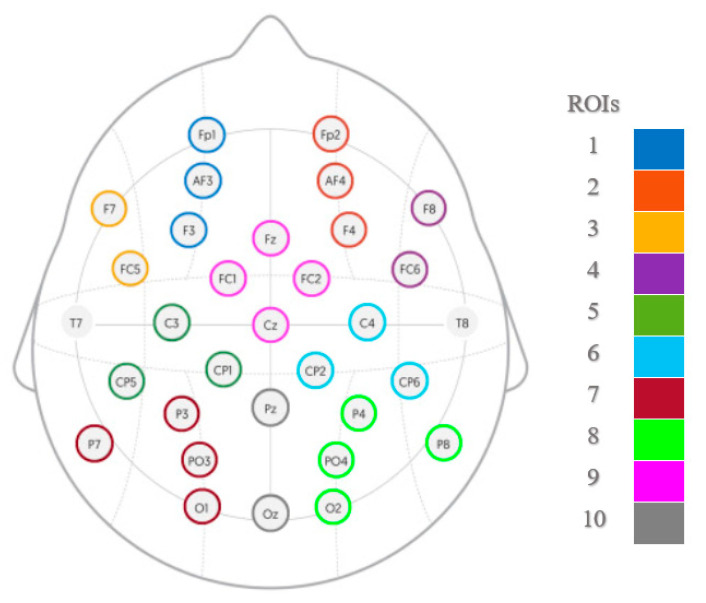
Electrode grouping into the regions of interest (ROIs): mid-frontal left (1) and right (2), left (3) and right (4) frontal, centro-parietal left (5) and right (6), parieto-occipital left (7) and right (8), fronto-central (9) and central parieto-occipital (10).

Ten regions of interest (ROI) were created according to Figure 3; electrodes within each ROI were averaged, and segments lacking information on at least one ROI (i.e., if all of the channels within that ROI have been removed during the artifact removal process) were excluded from the analysis, resulting in 314 segments retained for each emotional condition (5 × 314 segments in total). From the 30 s segments, eight 3 s non-overlapping windows were created (from 3 to 27 s) for each of the 10 ROIs. The dimensions of the final matrices were equal to 1570 × 10 × 1500 arrays. Finally, by filtering the separate rows of the matrices into different frequency bands for 1–4 Hz (delta), 4–8 Hz (theta), 8–12 Hz (alpha), 12–30 Hz (beta), and 30–45 Hz (gamma), five sub-band matrices (called observation matrices) were obtained for each emotional playing condition and corresponding neutral baseline.

### 2.5. Feature Extraction

Several features were extracted to be subjected to classification in order to discriminate between emotional performance conditions.

#### 2.5.1. Power Spectra

Spectral power features were calculated from each of the sub-band observation matrices using a Fast Fourier Transform (FFT) and by applying a Hanning tapering window. Five absolute values were computed corresponding to 5 sub-frequency bands of the delta, theta, alpha, beta, and gamma sub-bands, by averaging the power within these bands. Further, five more features were obtained as relative power within sub-bands by dividing the absolute values by the average power spectra of the entire band (1–45 Hz). Ten computed features for 10 ROIs (absolute and relative powers per five sub-bands) were placed in a row sequentially, producing a matrix containing 100 features per observation with the size of 1570 × 100 (one for emotional playing and one for their corresponding neutral baselines).

#### 2.5.2. Magnitude Square Coherence

Magnitude Square Coherence (MSC) was used to evaluate functional connectivity. MSC is a linear synchronization approach with values from 0 to 1 indicating the degree of correspondence between *x* and *y* at a given frequency of f; values closer to 1 indicate stronger linear dependency between two signals. The MSC is formulated as follows:

(1) $$\gamma_{xy}\left(f\right)=\frac{{\left|\langle S_{xy}\left(f\right)\rangle \right|}^{2}}{\left|\langle S_{xx}\left(f\right)\rangle \right|\left|\langle S_{yy}\left(f\right)\rangle \right|}$$

where $S_{xy}\left(f\right)$ is cross power spectral density, and $S_{xx}\left(f\right)$ and $S_{yy}\left(f\right)\;$are the power spectral density of the two different signals named *x*, y [14,15]. For all observations, the MSC was computed between each pair of ROIs, and the 10 × 10 connectivity matrices (CM) were created, where the array (*i,j*) illustrated the connection between regions numbered *i* and *j*; since the CM resulted from MSC was symmetrical relative to the diagonal line (array(*i,j*) = array(*j,i*)), the connectivity matrices were further turned into a 45-element row by placing their 10 × 9/2 arrays on the top or bottom of the diagonal line sequentially next to each other resulting in the observation matrices of 1570 × 45 (one matrix for emotional playing and one matrix for their corresponding neutral baselines per each of 5 sub-bands).

#### 2.5.3. Granger Causality

Granger Causality (GC) was used to calculate the effective connectivity. According to Granger Causality, if signal X “Granger-causes” signal Y, then using past values of X to predict Y should have less estimation error than using past values of Y alone to predict it [29]. GC is described as follows:

Assume the *y*(*t*) and *x*(*t*) be stationary time series and consider the ε(t) as the prediction error of *y*(*t*) calculated with lagged values of *y*(*t*) using the following autoregression model:

(2) $$y\left(t\right)=\varepsilon \left(t\right)+\overset{\infty }{\underset{i=1}{\sum }}a\left(i\right)\times y\left(t-i\right)$$

Following this, the model is recalculated as follows by including lagged values of *x*(*t*), and $\tilde{\varepsilon }\left(t\right)$ prediction error is calculated by considering the effect of lagged values of *x*(*t*) too:

(3) $$y\left(t\right)=\tilde{\varepsilon }\left(t\right)+\overset{\infty }{\underset{i=1}{\sum }}a\left(i\right)\times y\left(t-i\right)+\overset{\infty }{\underset{j=1}{\sum }}b\left(i\right)\times x\left(t-j\right)$$

*a*(*i*) and *b*(*j*) are regressive coefficients.

If the variance of $\tilde{\varepsilon }\left(t\right)$ is smaller than that variance of ε(t), then the GC will be 1 and *x*(*t*) ‘’Granger-causes’’ *y*(*t*); if the variance of $\tilde{\varepsilon }\left(t\right)$ is larger than that variance of ε(t) then GC value will be 0 and *x*(*t*) does not ‘’Granger-cause’’ *y*(*t*).

Similar to what has been done for MSC, the 10 × 10 CM was created for all of the observations by calculating the GC between each pair of regions. Then due to the unsymmetrical nature of CM resulting from GC, the connectivity matrices were turned into a 100-element row by placing all of their 10 × 10 elements sequentially next to each other, and the observation matrices of 1570 × 100 arrays were created (one matrix for emotional playing and one matrix for their corresponding neutral baselines for each of 5 sub-bands).

### 2.6. Feature Selection

Neutral baseline playing before emotional performance was included in the experiment in order to provide a baseline for emotional performances [30] and to reduce the dependence of the results on the mood of the subjects at any instant, as well as to eliminate inter-subject variability [11]. However, as the inclusion of neutral parts substantially extends the time required for experiment, and can also be boring for the performers, the real benefit of its inclusion needs to be evaluated. In this study, at the feature selection stage, we tested two approaches which are: (1) taking the neutral baseline into account while selecting features for classification further referred to as Normalized features, and (2) not taking the neutral baseline into account while selecting features for classification which will be referred to as Non-normalized features.

#### 2.6.1. Normalized Features

The feature selection process for the outcomes of power, MSC and GC, when neutral baseline playing was taken into account, involved two distinct phases. During the first phase, a two-sided Student’s *t*-test was applied to the data of each column in the feature matrix belonging to the emotional category and the same column in the baseline matrix to eliminate features that were not distinguishing between the baseline and emotional parts. The null hypothesis (both datasets are drawn from the same distribution) was rejected with *p*-values < 0.05. This step was ignored for 314 observations of neutral playing because no significant differences between the neutral baseline EEGs and the EEGs recorded during the expression of neutral emotions were expected. This step resulted in new feature matrices created from retained features meeting two criteria: (1) the ability to discriminate between the neutral baseline and the emotional playing and (2) being shared across conditions. During the second phase, to identify the features that were able to discriminate between at least two pairs of emotional conditions, a one-sided ANOVA was performed on the resulting feature matrices from the first step, and the features with a *p*-value < 0.01 were chosen as a set of final features.

#### 2.6.2. Non-Normalized Features

In this approach, the normalization using a Student’s t-test was omitted and only the one-sided ANOVA was performed on the outcomes of power, MSC, and GC analyses. The features that were able to distinguish between at least two pairs of emotional conditions (with a *p*-value < 0.01) were chosen to create the feature matrices.

### 2.7. Classification

A Support Vector Machine (SVM) was used for classification of the features selected. SVM is a supervised machine learning model that uses separating hyperplanes for discriminating classes. In this study, we used the “one-against-one” strategy which trains one SVM classifier for each pair of classes (C(5,2) = 10), then the new data is assigned to one of the classes with the majority of votes [31,32]. In terms of selecting the SVM kernel, the Radial Basis Function (RBF) kernel was chosen because it has been shown to be a better choice for practical applications that require real-time processing, in addition to its higher performance on data with higher dimensional space [33].

Three classifiers (5-fold cross validation, for Arousal aspect, Valence aspect, and emotional condition separately) were trained on each group of the features (power and features from MSC and GC) by labeling the data based on the instructions provided to the subjects [11] in Arousal, Valence, and emotional condition dimensions (including Distressed, Depressed, Neutral, Excited, and Relaxed, which are combinations of different Arousal/Valence levels). The utility of classifier was decided based on two major criteria: (1) the classifier is well-trained (not over-fitted nor under-fitted), (2) the classifier reaches the generalization based on all of the available data. When these criteria are met, the reported accuracies and precisions are considered reliable and repeatable. The first criterion was met when the training and test accuracies were fairly close to each other. For the second criterion, 5-fold cross-validation was used to evaluate the outcomes of the classifier. For this purpose, the 1570 observations in each of the feature matrices were first shuffled to combine the data of different subjects; then matrices were divided into 5 equal-sized non-overlapping sub-data with 314 observations. One of these sub-data (1 × 314) was used as the test data and the remaining (4 × 314) was used as the training data. This procedure was repeated 5 times, each time with new test data, and finally, the result is the average of all 5 accuracies.

The results of the non-binary classification were evaluated using confusion matrices where each column resembles the predicted category and each row indicates the actual category. The confusion matrices were created by subjecting the test data to the trained SVM classifier, then extracting the number of correct and wrong predictions.

## 3. Results

The outcomes of the behavioral assessement are presented in details in [11]. The outcomes of power, MSC, and GC analyses are reported in Supplementary Material, where power plots for each ROI, and MSC/GC connectivity maps are plotted (Figures S1–S3). The number of features selected for classification for power, MSC, and GC analyses on all frequency bands and both feature extraction approaches (Normalized and Non-normalized features) is presented in Table 1.

The accuracies of the classification performed for Arousal, Valence, and emotional conditions separately using two sets of features (Normalized and Non-normalized) are presented in Table 2. Both training and test accuracies were relatively close to each other, indicating that the models have achieved a good generalization with respect to the input data. The accuracies obtained from Non-normalized features were higher than the accuracies resulting from the usage of Normalized features. Moreover, MSC-based features showed the best discrimination accuracies for both Arousal and Valence dimensions, and for emotional conditions (85.57 ± 2.34, 84.93 ± 1.67, and 87.16 ± 0.55, respectively). In both sets of features, better classification results were obtained for Arousal compared to Valence dimension (for example: 76.98 ± 0.42 vs. 71.02 ± 2.04 for Normalized MSC, 85.57 ± 2.34 vs. 84.93 ± 1.67 for Non-normalized MSC).

**Figure 4:**
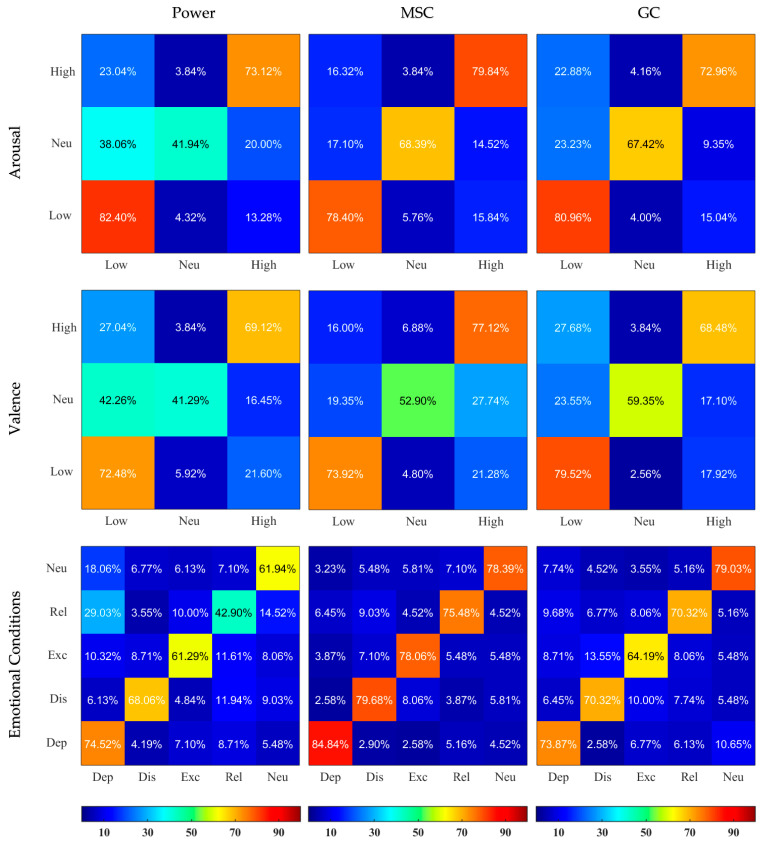
Confusion Matrices resulting from SVM classifiers applied on Normalized Power, MSC, and GC features and based on 3 different labelings. The X and Y axes represent the categories, the rows correspond to the actual class and the columns correspond to the predicted class. Dep—depressed, Dis—distressed, Exc—excited, Rel—relaxed, Neu—neutral. The predicted values are written inside the squares and are also color-coded according to the color bars provided.

**Figure 5:**
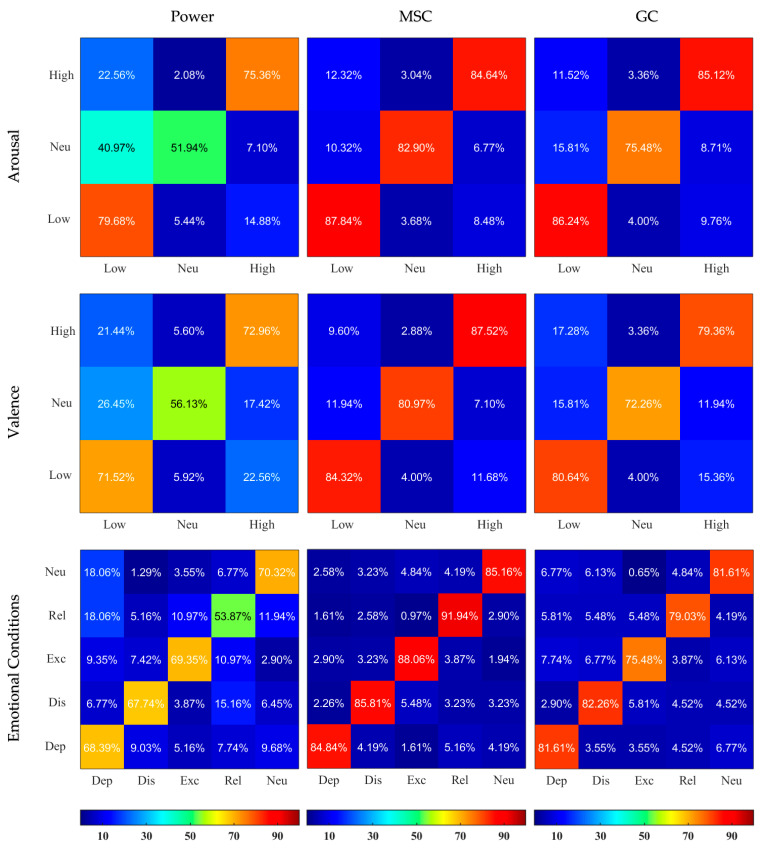
Confusion Matrices resulting from SVM classifiers applied on Non-normalized Power, MSC, and GC features and based on 3 different labelings. The X and Y axes represent the categories, the rows correspond to the actual class and the columns correspond to the predicted class. Dep—depressed, Dis—distressed, Exc—excited, Rel—relaxed, Neu—neutral. The predicted values are written inside the squares and are also color-coded according to the color bars provided.

The confusion matrices illustrating the outcomes of classification sensitivity separately for Normalized and Non-normalized features are depicted in Figure 4 and Figure 5. MSC-based features showed best outcomes for feature labeling of Arousal, Valence, and emotional conditions (for example using the Non-normalized features, 84.84% of actual Depressed data was classified as Depressed, while only 4.19%, 1.61%, 5.16%, and 4.19% were falsely classified as Distressed, Excited, Relaxed, and Neutral, respectively). Usage of Non-normalized features consistently lead to better discrimination compared to Normalized features. It was especially true for the MSC- and GC-based features, where the normalization greatly reduced the ability of classifiers to detect Neutral condition in Arousal and Valence classifications.

## 4. Discussion

We aimed to evaluate the utility of different EEG-based methods of feature extraction to discriminate between emotional conditions during active music playing. The measure of functional connectivity—MSC, and the measure of effective connectivity—GC—were of particular interest due to the low processing requirements and potential to be easily utilized for the development of a real-time BCMI system. The number of features was reduced utilizing two approaches of feature selection (normalizing by Neutral condition and without this normalization) and the SVM classifier was utilized to estimate the potential of the selected features to discriminate the emotional playing conditions.

We extracted features based on power and connectivity calculations, as these measures in our previous investigations showed some differences between emotional playing conditions [11,12], and were physiologically relevant. In this paper, we utilized two feature extraction approaches. First, we used neutral baseline playing to normalize the data. Although we expected the step of normalization to reduce individual variability and the impact of movement execution processes while performing, the normalization resulted in lower accuracies when classifying (Table 2). As the inclusion of the neutral baseline playing substantially increases the time of the experiment and has a negative impact on classification quality, we suggest that this step can be omitted in future studies.

All the achieved accuracies of classification were higher than chance level (1/3 ≈ 0.33 × 100 = 33% for Arousal and Valence, and 1/5 = 0.2 × 100 = 20% for emotional conditions) [34]. Importantly, the corresponding training and test accuracies were relatively close to each other, meaning that overfitting did not occur [35]. The best classification results were achieved using MSC-based features. MSC reflects functional connectivity with the assumption that when two things happen together, they might be related to each other [36]. The classification accuracies achieved with MSC-based features in the current study were comparable to those obtained for emotion classification while listening to music or observing emotional images in a study by Khosrowabadi et al. [36], who applied a Self-organizing map on MSC-based features. However, Shahabi et al. [16] reported slightly higher accuracies in comparison to this report when classifying responses to joyful, neutral, and melancholic music using DTF features; nonetheless, it should be stressed that DTF is not computationally efficient for BCMI development. Finally, Liu et al. [37] achieved lower accuracy levels in Arousal and Valence recognition with similar MSC-based features and a “K-nearest neighbor” classifier using responses to music from the DEAP dataset.

As can be seen from Table 1, the number of MSC-based features included in the classification was relatively equally distributed between the EEG sub-bands, pointing to the importance of the assessment of functional connectivity within a wide frequency range, and not limiting it only to certain ranges [38]. It contrasts the outcomes of GC results in the current study and earlier reports where brain entropy/signal complexity [39]/Granger Causality [12] within beta and gamma bands showed the most effects during improvised/emotional playing. Importantly, the classification of GC-based features while performing music resulted in accuracies very similar to those reported by Liu et al. while classifying music-induced emotions [40], and Guo et al. while detecting induced emotions in the data from the DEAP and SEED datasets using neural networks on GC features [41]. The summary of accuracies obtained in the above mentioned works is presented as Supplementary Material, Table S1.

For all features extracted, classification of the Arousal dimension and discrete emotional conditions showed better accuracy levels than classification of the Valence dimension. On the one hand, it is surprising, as previously Galvao et al. [38] and Bazgir et al. [42] showed very comparable classification outcomes for Arousal and Valence dimensions. On the other hand, our result confirms the fact that the arousal aspect (reflecting the degree of activation or intensity [43]) is important for emotional expressions—not only when listening to music, but also when performing.

However, it should be noted that classification outcomes for signals recorded while performing music and listening to music should not necessarily be comparable: as mentioned in the introduction, music performers do not necessarily experience the emotion that they need to communicate, thus brain activity can differ [25]. Nevertheless, the results mentioned above suggest that the balance between the physiological relevance of the features extracted and the computational efficiency of the classification methods should be considered when developing BCMI systems. The efficient online classification of musically expressed emotions for BCMI application would bridge major gaps between the fields of neuroscience, human-computer interaction, music computing, and the arts. More interdisciplinary work at this intersection is needed to target persistent challenges in the field, particularly a lack of application-specific tools for online feature detection and mapping for control which may be explored comprehensively within frameworks of music interaction.

## 5. Conclusions

In the present study, we assessed how well the power spectrum and two connectivity features—MSC and GC—could help to recognize emotional aspect of the musical performance. The connectivity-based features demonstrated acceptably high accuracies. In contrast, power-based features performed less accurately. Both MSC and GC are computationally efficient, and thus may be utilized to develop or improve the accuracy of a real time BCMI system.

## Figures and Tables

**Table 1:** Number of retained features after feature selection.

			Delta	Theta	Alpha	Beta	Gamma	Total
Power	Normalized	*t*-Test	12	15	14	19	20	80
		ANOVA	9	15	12	16	20	72
	Non-normalized	ANOVA	14	17	17	18	20	86
MSC	Normalized	*t*-Test	27	22	24	14	25	112
		ANOVA	26	22	23	8	25	104
	Non-normalized	ANOVA	39	37	36	33	40	185
GC	Normalized	*t*-Test	10	11	10	41	43	115
		ANOVA	8	10	7	35	38	98
	Non-normalized	ANOVA	22	30	30	68	76	226

**Table 2:** Accuracies (%) of SVM classifiers for Arousal, Valence and Emotional condition.

			Arousal	Valence	Emotional Conditions
Normalized Features	Power	Train	74.87 ± 0.79	69.28 ± 1.62	68.85 ± 1.32
		Test	70.64 ± 1.88	64.93 ± 2.56	61.74 ± 2.03
	MSC	Train	81.51 ± 1.90	76.93 ± 0.83	83.20 ± 0.60
		Test	76.98 ± 0.42	71.02 ± 2.04	79.29 ± 2.96
	GC	Train	79.61 ± 0.71	77.63 ± 0.71	76.65 ± 0.28
		Test	75.06 ± 1.26	71.08 ± 1.26	71.54 ± 1.34
Non-normalized Features	Power	Train	77.26 ± 0.55	72.00 ± 0.72	72.00 ± 1.68
		Test	72.43 ± 1.94	69.03 ± 0.39	65.93 ± 1.06
	MSC	Train	91.21 ± 0.45	90.05 ± 0.28	93.11 ± 0.13
		Test	85.57 ± 2.34	84.93 ± 1.67	87.16 ± 0.55
	GC	Train	88.68 ± 0.47	84.53 ± 0.87	85.63 ± 0.41
		Test	83.65 ± 1.28	78.46 ± 2.20	80.00 ± 1.51

## Data Availability

The data presented in this study are available on request from the corresponding author. The data are not publicly available due to privacy restrictions.

## Supplementary Materials

The following supporting information can be downloaded at: https://www.mdpi.com/article/10.3390/s23042252/s1, Supplementary Material: Power Spectra, MSC and GC analysis outcomes; a summary of outcomes of previous reports mentioned in the Discussion.

## References

[1] Minguillon J., Lopez-Gordo M.A., Pelayo F. (2017). Trends in EEG-BCI for Daily-Life: Requirements for Artifact Removal. Biomed. Signal Process. Control.

[2] Bonci A., Fiori S., Higashi H., Tanaka T., Verdini F. (2021). An Introductory Tutorial on Brain–Computer Interfaces and Their Applications. Electronics.

[3] Torres-Cardona H.F., Aguirre-Grisales C. (2022). Brain-Computer Music Interface, a Bibliometric Analysis. Brain-Comput. Interfaces.

[4] Miranda E.R. (2006). Brain-Computer Music Interface for Composition and Performance. Int. J. Disabil. Hum. Dev..

[5] Straebel V., Thoben W. (2014). Alvin Lucier’s Music for Solo Performer: Experimental Music beyond Sonification. Organ. Sound.

[6] Daly I., Williams D., Kirke A., Weaver J., Malik A., Hwang F., Miranda E., Nasuto S.J. (2016). Affective Brain-Computer Music Interfacing. J. Neural Eng..

[7] Bhattacharya J., Lee E.J. (2016). Modulation of EEG Theta Band Signal Complexity by Music Therapy. Int. J. Bifurc. Chaos.

[8] Sammler D., Grigutsch M., Fritz T., Koelsch S. (2007). Music and Emotion: Electrophysiological Correlates of the Processing of Pleasant and Unpleasant Music. Psychophysiology.

[9] Moon S.-E., Chen C.-J., Hsieh C.-J., Wang J.-L., Lee J.-S. (2020). Emotional EEG Classification Using Connectivity Features and Convolutional Neural Networks. Neural Netw..

[10] Lin Y.P., Wang C.H., Jung T.P., Wu T.L., Jeng S.K., Duann J.R., Chen J.H. (2010). EEG-Based Emotion Recognition in Music Listening. IEEE Trans. Biomed. Eng..

[11] Pousson J.E., Voicikas A., Bernhofs V., Pipinis E., Burmistrova L., Lin Y.P., Griškova-Bulanova I. (2021). Spectral Characteristics of EEG during Active Emotional Musical Performance. Sensors.

[12] Ghodousi M., Pousson J.E., Voicikas A., Bernhofs V., Pipinis E., Tarailis P., Burmistrova L., Lin Y.P., Griškova-Bulanova I. (2022). EEG Connectivity during Active Emotional Musical Performance. Sensors.

[13] Geethanjal B., Adalarasu K., Jagannath M. (2018). Music Induced Emotion and Music Processing in the Brain-a Review. J. Clin. Diagn. Res..

[14] Sakkalis V. (2011). Review of Advanced Techniques for the Estimation of Brain Connectivity Measured with EEG/MEG. Comput. Biol. Med..

[15] Cao J., Zhao Y., Shan X., Wei H.L., Guo Y., Chen L., Erkoyuncu J.A., Sarrigiannis P.G. (2022). Brain Functional and Effective Connectivity Based on Electroencephalography Recordings: A Review. Hum. Brain Mapp..

[16] Shahabi H., Moghimi S. (2016). Toward Automatic Detection of Brain Responses to Emotional Music through Analysis of EEG Effective Connectivity. Comput. Hum. Behav..

[17] Varotto G., Fazio P., Rossi Sebastiano D., Avanzini G., Franceschetti S., Panzica F. Music and Emotion: An EEG Connectivity Study in Patients with Disorders of Consciousness. Proceedings of the Annual International Conference of the IEEE Engineering in Medicine and Biology Society.

[18] Maggioni E., Arienti F., Minella S., Mameli F., Borellini L., Nigro M., Cogiamanian F., Bianchi A.M., Cerutti S., Barbieri S. (2021). Effective Connectivity During Rest and Music Listening: An EEG Study on Parkinson’s Disease. Front. Aging Neurosci..

[19] Marimpis A.D., Dimitriadi S.I., Goebel R. (2020). A Multiplex Connectivity Map of Valence-Arousal Emotional Model. IEEE Access.

[20] Koelstra S., Muhl C., Soleymani M., Lee J.-S., Yazdani A., Ebrahimi T., Pun T., Nijholt A., Patras I. (2011). Deap: A Database for Emotion Analysis; Using Physiological Signals. IEEE Trans. Affect. Comput..

[21] Mahmood D., Nisar H., Yap V.V., Tsai C.Y. (2022). The Effect of Music Listening on EEG Functional Connectivity of Brain: A Short-Duration and Long-Duration Study. Mathematics.

[22] McGuiness A., Overy K. (2011). Music, Consciousness and the Brain: Music as Shared Experience of an Embodied Present. Music and Consciousness: Philosophical, Psychological, and Cultural Perspectives.

[23] Leman M. (2008). Systematic Musicology at the Crossroads of Modern Music Research. Systematic and Comparative Musicology: Concepts, Methods, Findings.

[24] McPherson M.J., Barrett F.S., Lopez-Gonzalez M., Jiradejvong P., Limb C.J. (2016). Emotional Intent Modulates the Neural Substrates of Creativity: An FMRI Study of Emotionally Targeted Improvisation in Jazz Musicians. Sci. Rep..

[25] Gabrielsson A., Juslin P.N. (1996). Emotional Expression in Music Performance: Between the Performer’s Intention and the Listener’s Experience. Psychol. Music.

[26] Zangeneh Soroush M., Maghooli K., Setarehdan S.K., Motie Nasrabadi A. (2017). A Review on EEG Signals Based Emotion Recognition. Int. Clin. Neurosci. J..

[27] Peirce J., Gray J.R., Simpson S., MacAskill M., Höchenberger R., Sogo H., Kastman E., Lindeløv J.K. (2019). PsychoPy2: Experiments in Behavior Made Easy. Behav. Res. Methods.

[28] Delorme A., Makeig S. (2004). EEGLAB: An Open Source Toolbox for Analysis of Single-Trial EEG Dynamics Including Independent Component Analysis. J. Neurosci. Methods.

[29] Granger C.W.J. (1969). Investigating Causal Relations by Econometric Models and Cross-Spectral Methods. Econometrica.

[30] Gannouni S., Aledaily A., Belwafi K., Aboalsamh H. (2021). Emotion Detection Using Electroencephalography Signals and a Zero-Time Windowing-Based Epoch Estimation and Relevant Electrode Identification. Sci. Rep..

[31] Milgram J., Cheriet M., Sabourin R. “One against One” or “One against All”: Which One Is Better for Handwriting Recognition with SVMs?. Proceedings of the Tenth International Workshop on Frontiers in Handwriting Recognition.

[32] Güler I., Übeyli E.D. (2007). Multiclass Support Vector Machines for EEG-Signals Classification. IEEE Trans. Inf. Technol. Biomed..

[33] Patle A., Chouhan D.S. SVM Kernel Functions for Classification. Proceedings of the 2013 International Conference on Advances in Technology and Engineering (ICATE).

[34] Combrisson E., Jerbi K. (2015). Exceeding Chance Level by Chance: The Caveat of Theoretical Chance Levels in Brain Signal Classification and Statistical Assessment of Decoding Accuracy. J. Neurosci. Methods.

[35] Koehrsen W. (2018). Overfitting vs. Underfitting: A Complete Example. Towards Data Sci..

[36] Khosrowabadi R., Quek H.C., Wahab A., Ang K.K. EEG-Based Emotion Recognition Using Self-Organizing Map for Boundary Detection. Proceedings of the International Conference on Pattern Recognition.

[37] Liu J., Meng H., Nandi A., Li M. Emotion Detection from EEG Recordings. Proceedings of the 2016 12th International Conference on Natural Computation, Fuzzy Systems and Knowledge Discovery, ICNC-FSKD.

[38] Galvão F., Alarcão S.M., Fonseca M.J. (2021). Predicting Exact Valence and Arousal Values from EEG. Sensors.

[39] Dolan D., Jensen H.J., Mediano P.A.M., Molina-Solana M., Rajpal H., Rosas F., Sloboda J.A. (2018). The Improvisational State of Mind: A Multidisciplinary Study of an Improvisatory Approach to Classical Music Repertoire Performance. Front. Psychol..

[40] Liu J., Sun L., Liu J., Huang M., Xu Y., Li R. (2022). Enhancing Emotion Recognition Using Region-Specific Electroencephalogram Data and Dynamic Functional Connectivity. Front. Neurosci..

[41] Guo J., Fang F., Wang W., Ren F. EEG Emotion Recognition Based on Granger Causality and CapsNet Neural Network. Proceedings of the 2018 5th IEEE International Conference on Cloud Computing and Intelligence Systems, CCIS.

[42] Bazgir O., Mohammadi Z., Habibi S.A.H. Emotion Recognition with Machine Learning Using EEG Signals. Proceedings of the 2018 25th Iranian Conference on Biomedical Engineering and 2018 3rd International Iranian Conference on Biomedical Engineering, ICBME.

[43] Lindquist K.A. (2013). Emotions Emerge from More Basic Psychological Ingredients: A Modern Psychological Constructionist Model. Emot. Rev..

