# Supplementary material for: Assessment of Different Feature Extraction Methods for Discriminating Expressed Emotions during Music Performance towards BCMI Application
Source: Sensors (Basel). 2023 Feb 17;23(4):2252. doi: 10.3390/s23042252 (PMC9967688; doi:10.3390/s23042252)
Supplement: Supplementary file 1 [file sensors-23-02252-s001.zip › sensors-2157324-supplementary.pdf]

Supplementary Materials

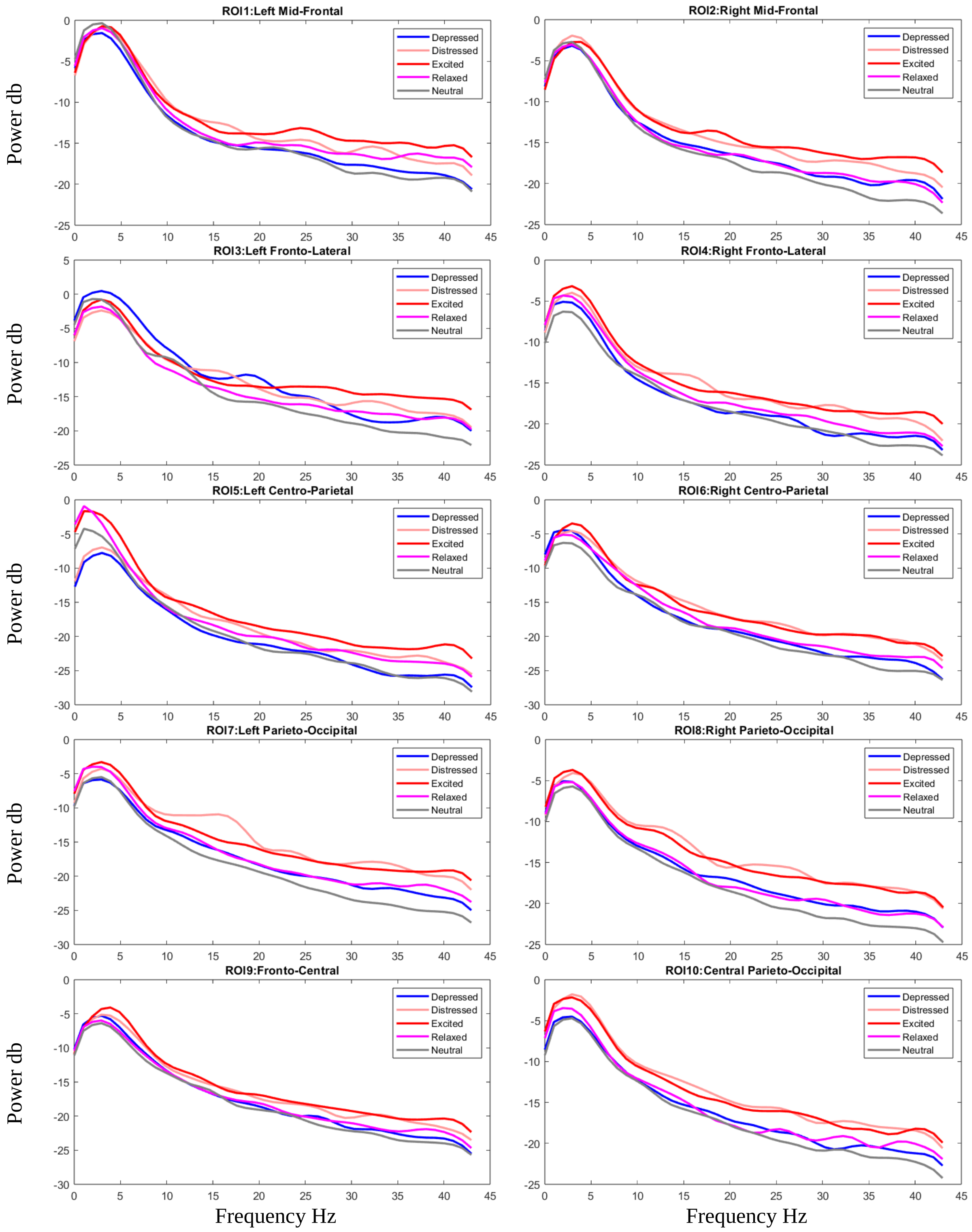

Figure S1. Power spectra for 4 ROIs for each emotional playing condition

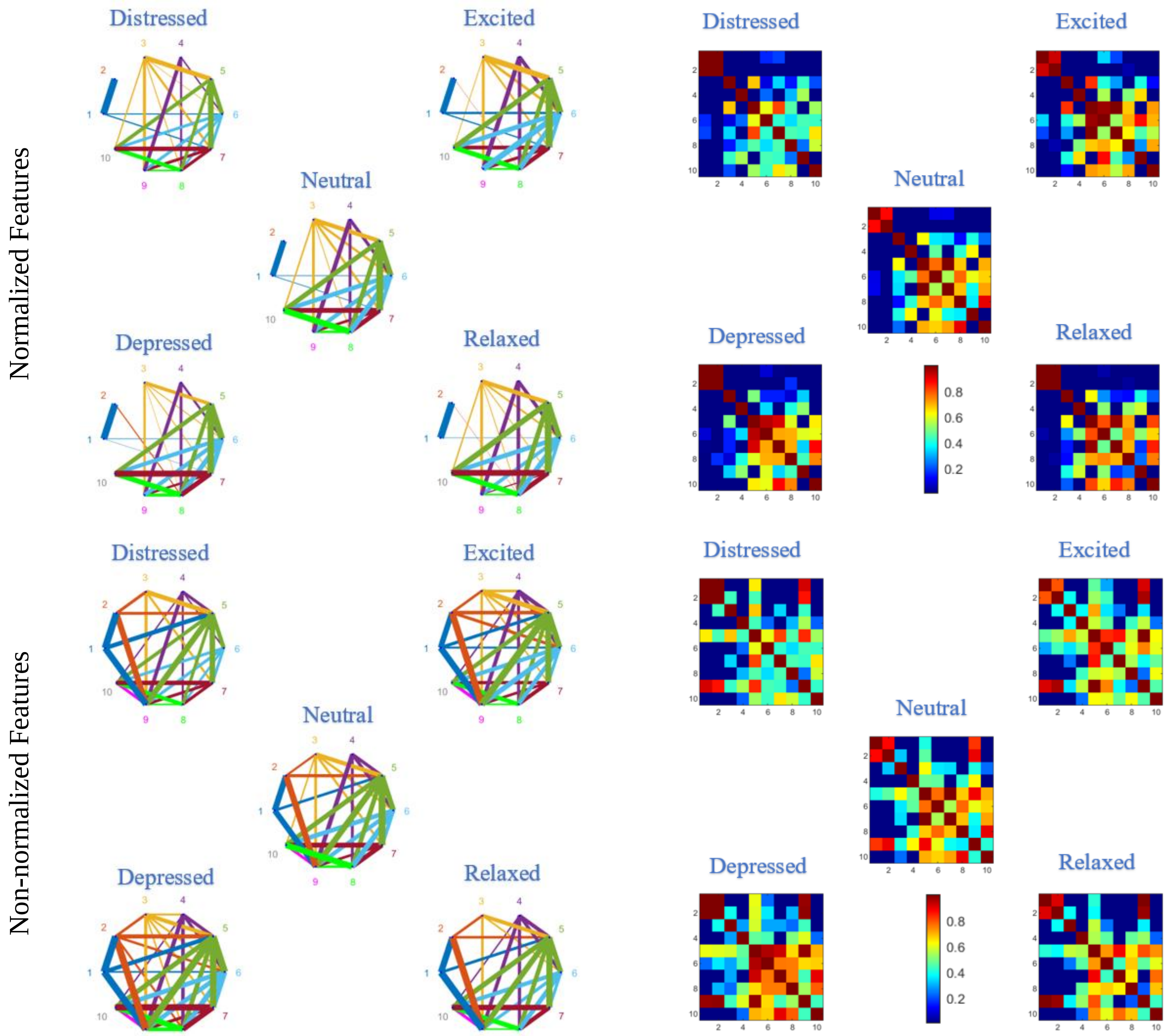

Figure S2. The connectivity plots (Left: Circular plot, Right: Raster plot) Resulted from MSC for five experimental conditions—neutral, depressed, distressed, excited, and relaxed performance instructions. ROIs coded by colors and numbers: mid-frontal left (1) and right (2), fronto-lateral left (3) and right (4), centro-parietal left (5) and right (6), parieto-occipital left (7) and right (8), fronto-central (9) and central parieto-occipital (10)). Left: lines connecting ROIs correspond to connections made between regions, and line widths indicate the strength of connections, while colors indicate no coding. Right: the colors of the intersection of each pair of regions code the strength of the connections.

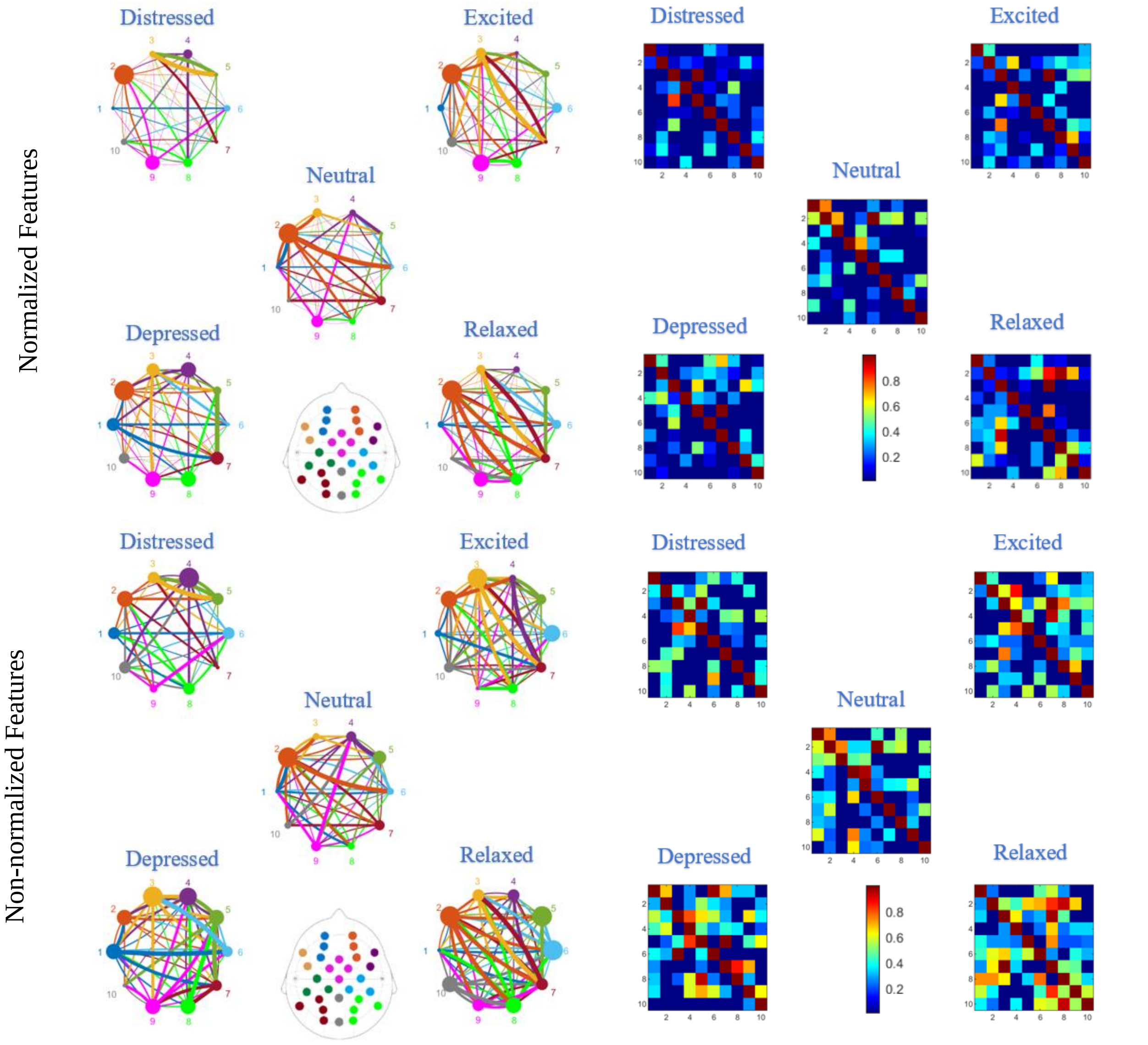

Figure S3. The connectivity plots (Left: Circular plot, Right: Raster plot) Resulted from GC for five experimental conditions—neutral, depressed, distressed, excited, and relaxed performance instructions. ROIs coded as numbers: mid-frontal left (1) and right (2), fronto-lateral left (3) and right (4), centro-parietal left (5) and right (6), parieto-occipital left (7) and right (8), fronto-central (9) and central parieto-occipital (10))- Left: Lines connecting ROIs correspond to connections made between regions, and line widths indicate the strength of connections, Line colors indicate the outputs of nodes with the same node color. Nodes are color-coded according to ROIs and the size of the circles reflects the number of outputs from each node - Right: the colors in the intersection of each pair of regions code the strength of the connections.

Table S1. A summary of outcomes of previous reports mentioned in the Discussion

Emotions	Stimulation	Recorded signals	Methods	Results	Ref
**Calm, Happy, Sad, Fear**	Emotional audio-visual	EEG, 8 Ch, 26 participants	MSC features, SOM for boundary selection, KNN classifier, 5-fold cross-validation	84.5% average classification accuracy for 4 classes	36
**Joyful vs Neutral, Joyful vs Melancholic**	Emotional music	EEG, 14 Ch, 19 participants	DTF features, Brute-Force feature selection, SVM classifier	80.43% Joyful vs Neutral- 93.7% for Joyful vs Melancholic	16
**Arousal-Valence Model**	Emotional music-video	EEG from DEAP dataset, 32 Ch, 32 participants	MSC, Time, Frequency, and Wavelet features, KNN and Random Forest classifiers, 10-fold cross-validation	71.23% and 69.67% classification accuracy under Arousal and Valence with the RF classifier on the fusion of features	37
**Arousal-Valence Model**	Emotional music-video	EEG from DEAP (32 Ch), AMIGOS (14 Ch), and DREAMER (14 Ch) datasets, 32, 40, and 23 participants	Spectral entropy, Wavelet energy and entropy, IMF energy and entropy features, KNN and Random Forest classifiers	89.8% average classification accuracy under Arousal and Valence, 84.4% average classification accuracy for the 4-class model (HAHV-HALV-LAHV-LALV)	38
**Arousal-Valence Model**	Emotional music	EEG, 32 Ch, 15 participants	Functional Connectivity features, Sequential backward selection algorithm for channel selection, Deep Neural Network classifiers	76.84% average classification accuracy under Arousal and Valence	40
**Arousal-Valence Model**	Emotional music-video	EEG from DEAP dataset, 32 Ch, 32 participants	GC features, sparse group lasso algorithm feature selection, KNN, SVM, CCN, and CaspNet Neural Networks (NN) classifiers	88.09% and 87.37% classification accuracy under valence and arousal emotion dimensions for Caspnet	41
**Arousal-Valence Model**	Emotional music-video	EEG from DEAP dataset, 32 Ch, 32 participants	Spectral Entropy features, PCA for dimension reduction, KNN, ANN, and SVM classifiers	91.3% and 91.1% classification accuracy under Arousal and Valence emotion for SVM	42
